# Resin-Bonded Fiber-Reinforced Composite for Direct Replacement of Missing Anterior Teeth: A Clinical Report

**DOI:** 10.1155/2011/845420

**Published:** 2011-09-20

**Authors:** Sufyan Garoushi, Lippo Lassila, Pekka K. Vallittu

**Affiliations:** ^1^Department of Biomaterials Science and BioCity Turku Biomaterials Research Program, Institute of Dentistry, University of Turku, Itainen Pitkakatu 4 B, 20520 Turku, Finland; ^2^Department of Restorative Dentistry & Periodontology, Institute of Dentistry, Libyan International Medical University, Benghazi, Libya

## Abstract

Missing anterior teeth is of serious concern in the social life of a patient in most of societies. While conventional fixed partial dentures and implant-supported restorations may often be the treatment of choice, fiber-reinforced composite (FRC) resins offer a conservative, fast, and cost-effective alternative for single and multiple teeth replacement. This paper presents two cases where FRC technology was successfully used to restore anterior edentulous areas in terms of esthetic values and functionality.

## 1. Introduction

Loss of anterior teeth is a common form of injury, particularly in children and adolescents. On the other hand, elderly people, who are retaining their teeth for longer period of time, have often advanced caries or periodontal diseases which may lead to extraction of teeth. Patients with lost anterior teeth require immediate attention for restoration of esthetic and functional reasons. The increased patient demand for tissue maintenance and esthetic, as well as the desire to reduce treatment costs, causes clinician to seek materials and techniques that enable minimally invasive and chairside (direct) fabrication on teeth replacement with fixed partial dentures (FPDs) [1, 2].

Over the last few years, the development of fiber-reinforced composite (FRC) has offered the dental profession the possibility of fabricating resin-bonded, esthetically good and metal-free tooth restorations for single and multiple teeth replacement. FRC-fixed partial denture (FPD) could be an alternative to metal frame resin-bonded-FPD, and also to full-coverage-crown-retained FPD and implant supported crowns [3, 4]. FRC, made of glass fibers, is the only existing esthetically acceptable material, which can be processed in mouth to the shape of a framework of a bridge, simultaneously adhere to the remaining tooth substance, and reach the adequate strength in terms of biting function of human. Many studies have focused on improvement of FRC FPD's strength [5, 6]. The most accepted concept to fabricate FRC FPDs is based on the use of continuous unidirectional glass (bundle) fibers in dimethacrylate-polymethylmethacrylate resin matrix as a substructure for the FPD [7]. With the FRC FPDs, there are two approaches on the use of the fibers: one is based on conventional tooth preparation and laboratory-made restorations, while the other is based on using the fibers in minimally invasive restoration (conservative) by direct or indirect fabrication. FRC systems enable the use of different retainer elements even in the same FPD (hybrid-type) [4]. For example, it is possible to create space for the occlusal support of the FRC framework by removing old filling or to make completely surface-retained restorations when clinical conditions allow correct designing of the FRC framework. In the dental literature, there are presently a limited number of clinical studies on the fiber-reinforced FPDs; however, based on those results, it is reasonable to expect FRC prostheses to have good longevity, especially with those made by direct technique [4, 8, 9].

This paper describes clinical cases of chairside-(directly) made FRC FPDs, which was used according to the principles of minimal invasiveness.

## 2. Clinical Report

### 2.1. Case 1

**Figure 1:**
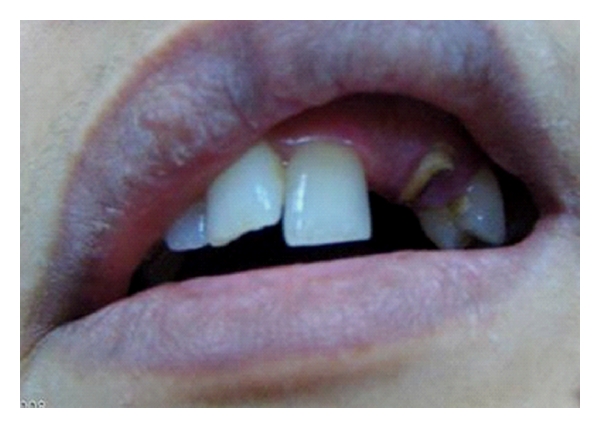
Frontal view of dentition of patient no. 1 with missing lateral incisor and endodontically treated canine.

Thirty-sex-year-old female patient had a chief complaint of esthetics because of a gab of missing upper left lateral incisor and crown of root canal-treated canine (Figure 1). The patient has normal horizontal and vertical overlap and canine-protected occlusion. After discussion with the patient, it became clear that the placement of an implant for the replacement of missing teeth was not possible due to high costs of the treatment. The fabrication of a conventional fixed partial denture was avoided and refused from patient in order to conserve the remaining tooth substance. Options for the conventional treatment with implants or crown-retained FPDs were remained open for the future. Directly made FRC FPDs were chosen in order to provide good esthetics, preserve tooth substance, and postpone more invasive treatments. The treatment was completed during one appointment. 

**Figure 2:**
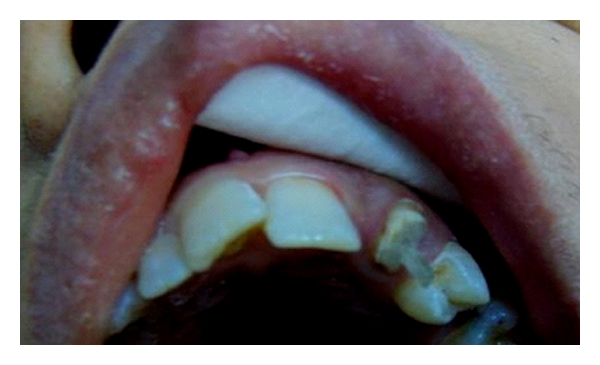
Fabrication of individual fiber post to the canine.

**Figure 3:**
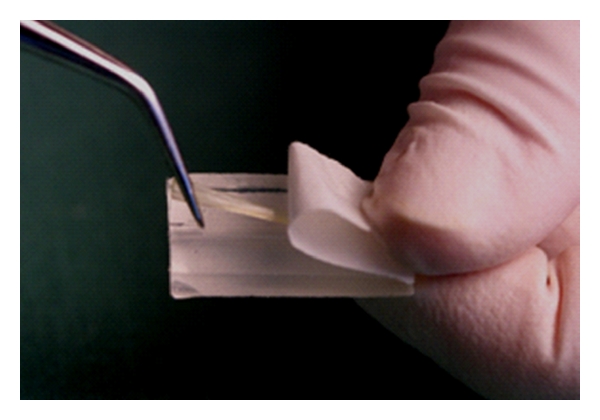
Resin impregnated fiber material is taken out from the package, which can be used as mold for adapting the fibers on tooth surface.

There was free occlusal space on the palatal surface of central incisor for FRC framework to be placed. Consequently, no cavity preparation for receiving vertical support for the bridge was needed. Cotton roll for isolation was used although the rubber dam is highly recommended. Guttapercha root canal filling at the upper left canine was removed using Gates Glidden burs up to size 4 for the total length of 7 mm (4000 cycles min^−1^ with water cooling). The root canal was prepared to receive a root canal post. The individually formed glass FRC post (everStick Post, StickTech Ltd, Turku, Finland) was prepared following the manufacturer's instructions (Figure 2). A bundle of preimpregnated glass fibers was cut to a length of 16 mm and spread from the ends for increasing the bonding surface area (Figure 3). The bundle was inserted in the canal and initially light polymerized with a hand light-curing unit (Optilux-501) for 20 s. Then the post was removed from the canal and additionally light polymerized for 40 s. The surface of the FRC post system was then wetted with resin (Stick Resin) and protected from any light source by a light proof box (3 M-ESPE, Germany) until cementation. Cement (ParaCem Universal, Switzerland) was placed on the post and the post was seated and extra cement was removed. The FRC framework was extended from the palatal surface of premolar to palatal surface of central incisor passing by the FRC post of the canine. 

**Figure 4:**
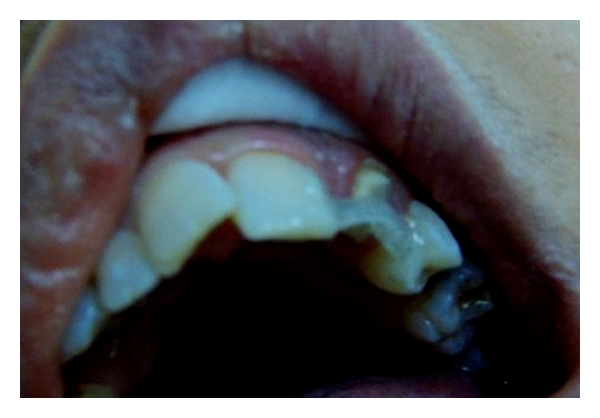
Appearance of the FRC framework with a layer of flow composite between FRC and tooth. Note labial positioning of the framework on the root canal post of canine.

**Figure 5:**
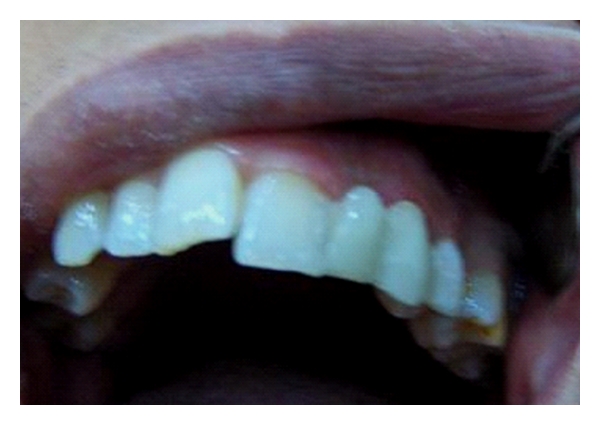
Frontal view of the final restoration.

**Figure 6:**
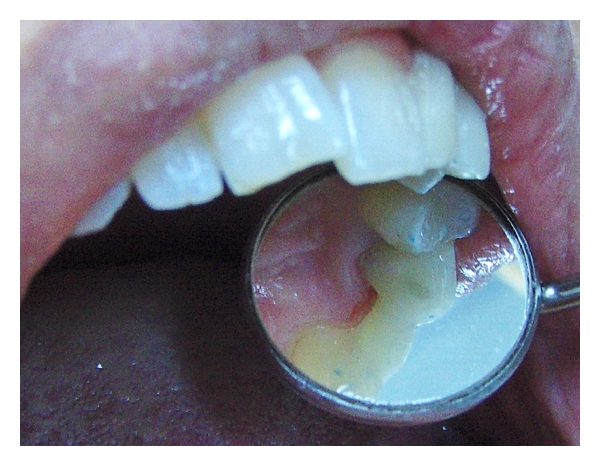
Palatal view of the final restoration.

After application of acid etching (37% phosphoric acid gel), the gel was rinsed thoroughly and gently air dried. Adhesive resins were applied according to the manufacturer's instructions (Scotchbond multipurpose adhesive, 3M ESPE, USA) to tooth surface. Flowable composite resin (Stick Flow, StickTeck Ltd, Turku, Finland) was applied on the bonding surfaces prior placing the resin impregnated fibers (everStick). The flow composite was not light cured before fibers were pressed tightly against the tooth surface using a transparent silicone package (mold) of the fibers. The resin impregnated fibers were light cured initially through the silicone mold. The purpose of the flow composite was to seal the space between the fibers and the enamel surface. The fiber framework was polymerized two times for 40 seconds (Figure 4). Fiber framework was fully covered with a thin layer of flow composite resin, and pontic was built up layer by layer using hybrid-type particulate filler composite resin. Successful chemical bond between fiber framework and veneered composite was achieved by curing. The shade of final veneered composite resin was selected using composite shade guide, and occlusion was carefully adjusted with articulating paper (Figures 5 and 6).

The occlusion was adjusted carefully to avoid any primary or premature contacts or traumatic occlusal forces to the restored teeth. The treatment outcome has been followed over three years without existence of any kind of serious problem.

### 2.2. Case 2

**Figure 7:**
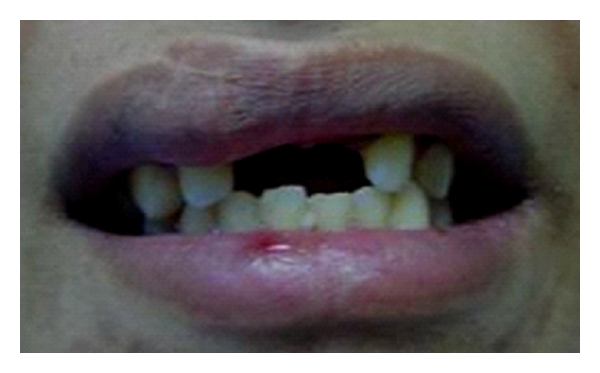
Frontal view of the dentition of patient no. 2.

Fourteen-year-old male patient lost his upper central incisors due to trauma by an accident (Figure 7). After discussion with the patient's father, it became clear that the placement of a single implant for the replacement of missing tooth was not possible due to patient age and high costs of the treatment. Also, young age of the patient would have been a clear contraindication for an implant treatment. The fabrication of a conventional fixed partial denture was avoided in order to conserve the tooth substance because of patient's young age. The missing teeth were planned to be replaced with an implant-retained crowns later on. Directly made FRC FPDs were chosen in order to provide good esthetics, preserve tooth substance, and postpone the final decision on the prosthetic treatment. The treatment was completed during one visit appointment. 

**Figure 8:**
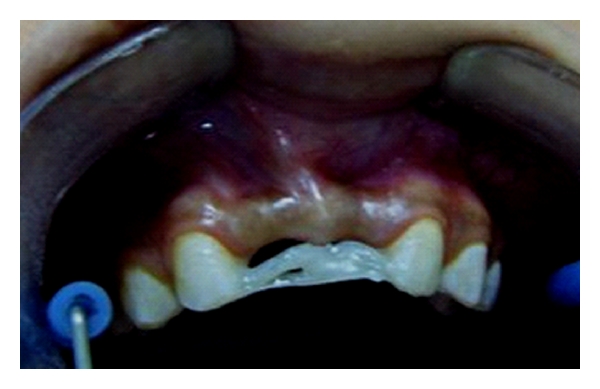
Appearance of fiber framework made two everStick C&B fiber bundles which covered large bonding areas on lateral incisors.

**Figure 9:**
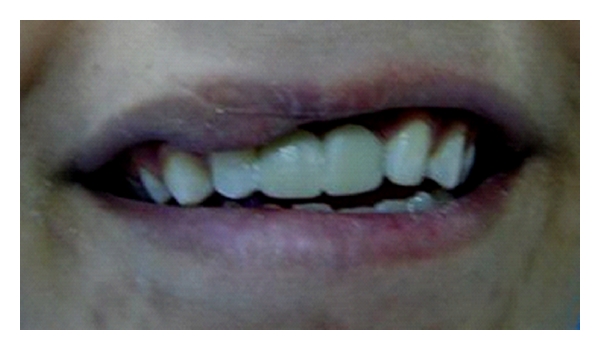
Final view of the restoration having FRC framework veneered with filling composite resin.

As described in the first clinical case, after etching and applying bonding agent, fibre-framework was extended between the palatal surfaces of lateral incisors (Figure 8). Fiber-framework was covered with a thin layer of flow composite resin, and pontic was built up by using particulate filler composite resin. The final step was the adjustment of occlusion and contouring and finishing the restoration (Figure 9). The outcome has been monitored over one year with no evidence of problems.

## 3. Discussion

Laboratory-made surface-retained resin-bonded prostheses made of metals are commonly supported and bonded from one end in order to reduce the number of debondings. In the case of surface-retained FRC prostheses, the framework can be supported from both ends because of better bonding characteristics and biomechanical flexibility of the FRC framework [4]. The flexibility allows abutment teeth to move without stressing the cement-framework interface in function, and loosening the prostheses. However, in the case of abutments with increased mobility, it is recommended to support also resin-bonded FRC FPD from one end only. In the cantilever designs, special care has to be taken to ensure adequate design-based rigidity of the FRC framework to resist bending forces by biting function. Adequate rigidity is obtained by increasing the cross-sectional diameter of the connector. Fibers of the framework should cover as large surface as possible on the abutments and, in the anterior area, should be placed close to the incisal edge to eliminate the dislodging forces [10].

Although resin-bonded FRC FPDs are most commonly used in the anterior and premolar regions, rather than molar region, recent laboratory investigations have suggested that optimally designed FRC FPD made on nonprepared abutments can provide even higher load-bearing capacity for the FPD than conventional porcelain-fused to metal FPD can provide [11]. Thus, the development of the FRC materials and technologies may allow alternatives also for directly made molar replacements. 

The FRC framework is intended to be fully covered by veneering composite in order to obtain a polishable and tooth-coloured surface. Special attention needs to be paid to the interproximal regions. If the FRC framework is not properly covered by veneering composite, the darkness of the oral cavity can be transmitted through the connectors and can cause esthetic problems [10].

The composition of the polymer matrix and fiber orientation has the major role in bonding ability and durability of veneered composite to the FRC framework or resin luting cement. It has been concluded that preimpregnation of the fibers with the light-polymerizable dimethacrylate resin system containing linear polymer phases is of importance to optimize the interfacial adhesion of FRC framework to composite veneer. Using a combination of dimethacrylate monomer resin and linear polymer, which forms semi-interpenetrating polymer network (semi-IPN) after being polymerized, offers better bonding site for veneered composite by means of interdiffusion bonding [12–14]. Recent laboratory studies showed that bond strength of directly fabricated FRC FPD to the tooth surface is as good as particulate filler composite [15]. 

From clinical point of view, there is a lack of long-term clinical research of FRC prostheses. However, the longitudinal studies reported general failure rates between 5% and 16% over periods up to 4-5 years [4, 9, 16]. These finding were demonstrated for prostheses with both extracoronal and intracoronal retainer designs, but only for patients who did not exhibit sever parafunctional habits. Van Heumen et al. showed a survival rate of 64% after 5 years follow-up of 3-unit anterior FRC prostheses made with the materials and techniques used in late 1990s [17]. One study reported a much higher failure rate of 40% over a 3-year period [18]. The recent clinical data, on the semi-IPN resin matrix FRC FPDs made directly in patients mouth, suggest high survival percentages (>96% at five years), which reflects material development and learning of fabricating FRC FPDs [19]. Most common failures in FRC FPDs reported in the earlier studies were delamination of veneering composite at pontic area, which are normally easy to be repaired in patients mouth. The current designing principles enable to fabricate FRC FPD to eliminate known risks for technical failures. 

As conclusion, the combination of filling composite veneer, adhesive system, and FRC framework has introduced a new generation of metal-free direct teeth replacement. The most recent fabrication principles need to be followed to ensure clinical success of the restorations.
